# The Contribution of Nearshore Fish Aggregating Devices (FADs) to Food Security and Livelihoods in Solomon Islands

**DOI:** 10.1371/journal.pone.0115386

**Published:** 2014-12-16

**Authors:** Joelle A. Albert, Doug Beare, Anne-Maree Schwarz, Simon Albert, Regon Warren, James Teri, Faye Siota, Neil L. Andrew

**Affiliations:** 1 WorldFish, Honiara, Solomon Islands; 2 WorldFish, Penang, Malaysia; 3 School of Civil Engineering, The University of Queensland, St. Lucia, Australia; 4 Solomon Islands Ministry of Fisheries and Marine Resources, Honiara, Solomon Islands; 5 Australian National Centre for Ocean Resources and Security, University of Wollongong, Wollongong, Australia; Leibniz Center for Tropical Marine Ecology, Germany

## Abstract

Fish aggregating devices, or FADs, are used widely in developing countries to concentrate pelagic fish, making them easier to catch. Nearshore FADs anchored close to the coast allow access for rural communities, but despite their popularity among policy makers, there is a dearth of empirical analysis of their contributions to the supply of fish and to fisheries management. In this paper we demonstrate that nearshore FADs increased the supply of fish to four communities in Solomon Islands. Estimated total annual fish catch ranged from 4300 to 12 000 kg across the study villages, with nearshore FADs contributing up to 45% of the catch. While it is clear that FADs increased the supply of fish, FAD catch rates were not consistently higher than other fishing grounds. Villages with limited access to diverse or productive fishing grounds seemingly utilized FADs to better effect. Villagers believed FADs increased household income and nutrition, as well as providing a source of fish for community events. FADs were also perceived to increase intra-household conflict and reduce fishers' participation in community activities. FADs need to be placed within a broader rural development context and treated as another component in the diversified livelihoods of rural people; as with other livelihood options they bring trade-offs and risks.

## Introduction

Coastal fisheries are central to the rural economies and food supply of Pacific Island Countries and territories (PICTs), supplying daily food and serving as one of the few sources of cash for villagers and coastal people [1]. In a widely cited publication from 2009, Bell and co-authors predicted that coastal fisheries in many countries in the region would not be able to provide enough fish to meet peoples' needs by 2030. If correct, this bleak conclusion adds to the broader global narrative about the future of fish (e.g. [2]) and should have profound policy and development assistance consequences. In the popular imagination, as much as in considered policy circles, food insecurity is not a challenge that usually emerges when contemplating the future of the Pacific peoples [3].

In order to avoid this projected supply deficit, sources of fish need to diversify and the management of coastal fisheries will need to improve. Many PICTs own large tuna resources, and bringing tuna to rural communities may play a major role in alleviating the fish shortages [4]–[6]. While policies and natural resource management strategies that aim to substitute the unsustainable harvest of reef fish with an increased domestic supply of currently plentiful tuna are appealing, they would require profound structural changes to tuna value chains. Such changes may include landing a greater proportion of the oceanic commercial catch and also changes in processing and marketing of tuna (Bell *et al.* unpublished data). Diversifying catches from nearshore resources, and better management of those resources, must remain a central policy prescription for improving the food security of rural communities in the short-medium term.

Fish aggregating devices, known as FADs, are used widely in tropical and subtropical waters to concentrate pelagic fish [7]–[9], making them, at least in theory, easier to catch. Nearshore FADs are anchored to the sea floor, close to the coast to allow access for coastal communities, including by paddle canoe [6]. FADs are not a new innovation and have been used, in one form or another, in most PICTs for a long time, although until recently, mostly in the industrial sector [10], [11]. In 2000, Désurmont and Chapman reviewed the deployment of anchored FADs and suggested that FADs had been universally successful, at least in terms of aggregating fish. The authors noted that anything floating in the water actually seemed to be effective in persuading fish to congregate. Attribution of “success”, however, is complicated, and attracting fish is not the only requirement [11].

FADs are popular among policy makers seeking options to diversify the supply of fish. Belief in the effectiveness of FADs has been such that investments have been dominated by practical issues about FAD design and deployment [10], [12] rather than quantifying realized benefits. The few analyses that exist indicate that the value of fish caught (during a typical deployment timespan) may be up to seven times the cost of FAD construction and deployment [13] with return on investment dependent on FAD longevity [14]. Furthermore, catch rates are often higher and fuel costs lower, compared to fishing in the open ocean where aggregations of pelagic fish can be difficult to find [5], [10], [13], [15], [16].

These analyses notwithstanding, if nearshore FADs are to become more widespread, policy makers need a more robust empirical analysis of their contribution to food security in the region. Viewed though a rural development lens, FADs can be seen as just another intervention in a complex social landscape, in which pre-existing issues around land tenure (including nearshore waters) and access rights can make the logic of FAD deployment and use more difficult. Their deployment, in the absence of a broader understanding of the local context, often causes them to fail. Vandalism, for example, is a recurrent problem, and many FADs often do not remain in the ocean for more than a few weeks or months [12], [14], [17].

This paper contributes to filling the information deficit in the use of nearshore FADs as fisheries management tools and as contributors to food security in the Pacific region. The work reported here was part of a larger collaboration between Solomon Islands Ministry of Fisheries and Marine Resources (MFMR), Secretariat of the Pacific Community (SPC), University of Queensland (UQ) and WorldFish. The long-term goal of the collaboration was to develop the foundation for a national program of nearshore FADs for Solomon Islands. An important step in this process was to test whether FADs provide benefits to both fishers and their communities, and to better understand the perceptions and realities of community members to FAD deployment and use. The key question explored by this study is whether the presence of FADs near a fishing village increased either the efficiency of fishing (catch rates) or the total amount of fish landed. We describe FADs deployed in four communities in Solomon Islands and compare the dynamics of fishing those FADs with benthic and pelagic fishing.

## Methods

**Figure 1:**
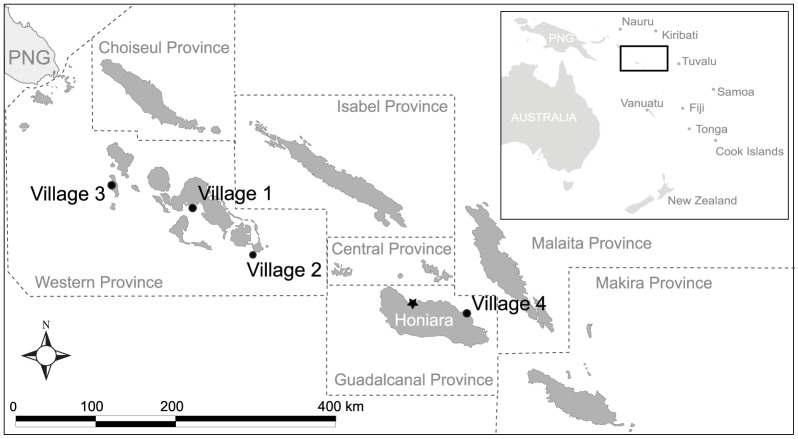
Map of Solomon Islands showing the location of the four study sites.

This paper focuses on four villages in Solomon Islands. A broader study deployed 21 FADs at 13 locations between March 2011 and October 2012. At each village, fishing catch and effort were monitored by trained community monitors, however, due to variable dedication of the monitors, and fishers' willingness to participate, adequate records were limited to four villages. Detailed information on FAD design and location for each of the study villages are provided in S1 File and S1 Table. Three of the villages were located in Western Province and one village was located in Guadalcanal Province (Fig. 1).

### Ethics statement

Research clearance, which included ethics clearance, was provided through an MOU that WorldFish has with the Solomon Islands Government and by WorldFish under its Code of Ethics for working with people (2009). Interviewees gave verbal consent to participate in the study and if verbal consent was not given the interview did not proceed. Written consent was not sought because of low levels of literacy. WorldFish approved the verbal consent process. Village names are not provided to maintain community confidentiality, and are referred to as Villages A, B, C and D.

### Study sites

Village A was located within Roviana Lagoon in Western Province where fishers target a complex reef system of mangrove, shallow seagrass beds, lagoon, reef, and deep ocean passages. About ∼400 people live in Village A and have been actively involved in community-based marine resource management activities for >10 years. Nearshore FADs were deployed near this village at a depth of 450 m on two occasions. The initial FAD was deployed 4.5 km from the village and was vandalized after 15 days. A second FAD was deployed closer to the village (2.7 km away) to enable improved access and security and lasted >3 months. Paddling distance to this second FAD was similar to the median distance to other non-FAD fishing grounds.

Village B, a remote community on the southern coast of Gatokae Island in Marovo Lagoon, has a small population of ∼120 people. The fishing environment at Village B was characterized by a narrow fringing reef, dropping into deep (>300 m) oceanic waters. Two FADs were deployed near this village, as part of the community's marine resource management activities. The FADs were located ∼1 km and 4.5 km from the village, at a depth of ∼400 m. Both FADs remained in the sea for almost 12 months before being lost during rough seas. The nearest FAD (1 km) was much closer for fishers to access, compared to the median distance to other fishing grounds.

Village C, on the western coast of Ranongga Island, had a population of ∼340 people. The fishing environment at Village C was characterized by a narrow fringing reef, exposed to the oceanic waters with deep-sea fishing grounds located relatively close to the coast. Fishers from this village also targeted offshore industrial FADs located >8 km from the village. Two nearshore FADs were deployed near this village at 450 m depth. One FAD was within 2 km of the village, while the second was deployed primarily for access by a neighboring village (7.5 km away). The closest FAD was moored at similar distances to other fishing areas. The two FADs remained in the water for >2 years.

Village D had ∼280 people and was located on eastern Guadalcanal Island. This stretch of coastline had few reefs - the only reef systems accessible by paddle canoe were located on a small peninsula adjacent to the village, and at a set of small islands more than 7 km from the coast. Fishing opportunities were primarily in open oceanic waters and rivers. Three FADs were deployed in the same location (at 265 m depth) on three separate occasions. The first FAD was lost to suspected vandalism and the second broke free in rough seas. The third deployment used a modified design and was more successful, lasting for more than 2 years. FADs in Village D were moored in the shallowest depths of all sites in the study, and were located furthest from the coast (7.3 km) and further than the median distance to other fishing grounds for this village.

### Catch and effort sampling

Fishing catch and effort was monitored at the study villages by trained community fishers, using modified sampling protocols developed by SPC [18]. Monitoring was done one day per week on a typical fishing day designated by the community. On the designated day, all fishing trips in the village were recorded, with non-FAD and FAD fishing recorded as separate trips. For each fishing trip the following data were recorded: village name, recorder name, fisher name, date, type of boat used, departure and return time, time spent fishing, number of fishers (disaggregated by sex), quantity of fuel used if applicable, fishing site name, species caught, total number and weight of each species, the fishing gears used, and finally, the intended purpose of the catch (i.e. for food, sale or bait). At each village, sampling began prior to the FAD being deployed and continued for as long as possible after deployment (typically 6 to 12 months).

### Fishing method and sea safety training

Troll line fishing has been the most common means of fishing FADs in the Pacific region, yet such gears are only targeting the fish near the surface [19]. Larger fish, and those typically found at deeper depth around FADs may be underutilized by fishers due to limitations in fishing gear and techniques [11], [19]–[21]. In response to this, SPC has developed fishing gear and method training specific for FADs [21] and have undertaken trainings across the region in association with FAD deployments [19]. As part of this broader project the opportunity arose for WorldFish, MFMR and some community members to join a fishing method and sea safety training workshop facilitated by SPC. A village member from Village A and Village B joined in this workshop, with the notion that they would train other fishers in their respective villages. During training it was apparent that some of the methods used were not suitable to the fishing gears and boats available to rural Solomon Island fishers, and subsequently a modified fishing method training was developed. Village-based fisher training using the modified methods were undertaken at villages C and D.

### Catch and effort analyses

**Figure 2:**
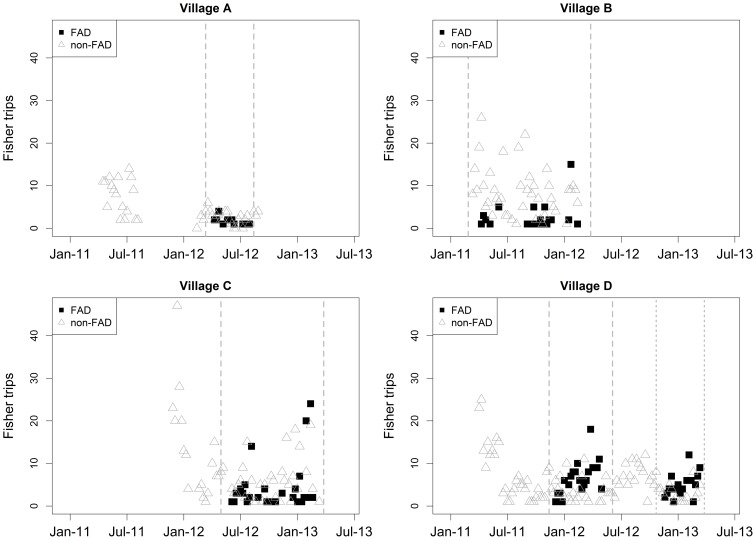
Number of fishing trips per week recorded during monitoring for FAD and non-FAD fishing at the four study villages. The vertical dashed lines represent the duration of the FADs in the water and the data used for analysis.

The key question explored by this study is whether or not the presence of FADs near a fishing village increased either catch rates or the total amount of fish landed. As in most small-scale tropical fisheries, a single fishing trip may use several gear types and most fishing trips caught a diversity of fishes. The sampling strategy was not designed to deal with this complexity, so in order to compare catch rates and catches between FAD and non-FAD fishing at any given site, we based our analysis on the entire catch from each fishing trip. To exclude any influence of seasonality on catch and effort, only data recorded during the period when a nearshore FAD was actually present at each village were used (Fig. 2). Fishing trips from Village C to offshore industrial FADs (set to aggregate tuna and other larger fishes) were excluded from the analysis.

Catch rates, in weight or number of fish caught per fisher per hour of fishing (fisher^−1^ hr^−1^) for each village were calculated as arithmetic means (± S.E.) for all fishing trips over the specific sampling period for each village (Table 1). Catch rates for different fishing gears were calculated the same way, but only using those fishing trips that solely used the gears of interest (i.e. fishing trips that used multiple gears were excluded from the analysis).

**Table 1:** Number of fishing trips (trips) and mean catch rates (± SE) by weight (CPUE_KG_) and number of fish (CPUE_N_) recorded for all gears, troll line and drop stone fishing at the four study villages.

		All gears		Troll line		Drop stone	
		FAD	Non-FAD	FAD	Non-FAD	FAD	Non-FAD
Village A	Trips	18	306	18	21	0^a^	24^a^
	CPUE_KG_	2.96±0.37	2.1±0.36	2.96±0.39	3.79±1.21	NA	NA
	CPUE_N_	11.3±1.85	5.94±0.73	11.3±1.85	1.73±0.36	NA	NA
Village B	Trips	41	232	37	7	4^a^	74^a^
	CPUE_KG_	1.68±0.41	2.16±0.16	1.78±0.45	2.07±0.75	NA	NA
	CPUE_N_	7.74±1.53	5.66±0.54	8.49±1.65	3.36±2.33	NA	NA
Village C	Trips	113	416	75	16	27	146
	CPUE_KG_	2.53±0.29	2.07±0.19	2.02±0.18	3.24±1.39	4.13±1.03	1.89±0.15
	CPUE_N_	2.52±0.23	2.78±0.24	2.88±0.30	3.11±1.58	1.63±0.37	2.34±0.11
Village D	Trips	207	485	151	70	50	50
	CPUE_KG_	1.04±0.06	0.87±0.06	1.03±0.07	0.84±0.08	1.11±0.11	0.94±0.11
	CPUE_N_	4.96±0.30	2.53±0.22	6.11±0.36	2.87±0.34	1.49±0.13	2.08±0.17

a Insufficient records for statistical analysis.

### Fish usage

Information collected on the proportion of fish caught and used for food was used to calculate the proportion of fish that were consumed by fisher families and scaled up to estimate consumption at the village level. Note that village-scale consumption was underestimated because fish purchased by community members at markets were not included in the analysis.

### Annual fish catch in each village

The sampling design allowed total daily catch from FADs and all other fishing areas to be estimated by village, and the proportion of the catch consumed by fisher families. These data were extrapolated to provide a preliminary estimate of the annual contribution of nearshore FADs to the supply and consumption of fish to the village. For this extrapolation, the offshore industrial FAD catches from Village C (excluded from the catch rate analysis) were included within the non-FAD dataset to provide a more complete representation of total fish catch. The number of fishing days per week at FAD and non-FAD fishing areas were derived from key informant interviews. Total daily catch (for FAD and non-FAD fishing) was multiplied by the respective mean number of FAD and non-FAD fishing days per week (for the corresponding village), and then by 52 weeks. The estimated annual fish catch reported in Table 2 is the mean (± S.E.) of the extrapolated daily catch for each village during the sampling period. Annual estimated fish consumption was calculated the same way, but using the proportion of daily catch recorded as being for consumed by the fishers and their families.

**Table 2:** Estimated annual fish catch and annual fish consumed (mean ± SE) from FAD and non-FAD fishing at the four study villages.

**Annual fish catch by weight (kg)**				
	Village A	Village B	Village C	Village D
FAD	1750±85	1340±76	4290±189	3780±69
Non-FAD	3380±93	2920±36	7690±158	4670±118
Total fish catch (kg)	5130	4260	11 980	8450
Contribution of nearshore FAD (%)	34.1	31.5	35.8	44.7
**Annual fish consumed (kg)**				
	Village A	Village B	Village C	Village D
FAD	1360±63	1010±58	3680±170	1490±40
Non-FAD	3010±90	2750±36	6730±146	1010±26
Total fish consumed (kg)	4370	3760	10 410	2500
Contribution of nearshore FAD (%)	31.1	26.6	35.3	57.8

### Villager perceptions of nearshore FADs

Key informant interviews were conducted at the four villages to gain insight into the social and economic factors associated with having access to a nearshore FAD. In total 69 interviews were conducted across the study villages, representing 30–59% of village households (S2 Table). Key informant interviews at Village B were undertaken under a related project and used different questions, so this village was excluded from this analysis. Results from four open-ended questions posed at villages A, C and D are presented in this paper:

1. Have there been any benefits of the nearshore FAD for your family; if so what?
2. Have there been any negative aspects of the nearshore FAD for your family; If so what?
3. Have there been any benefits of the nearshore FAD for your community; if so what?
4. Have there been any negative aspects of the nearshore FAD for your community; if so what?

Individual responses related to the benefits and negative aspects of the nearshore FADs were categorized. The ‘benefit of FADs’ categories included using fish for: fundraising and feasts, income (fish sold at markets), improved nutrition (including responses of an increase in fish consumption), improved access to fish, improved food security, and other social dimensions such as building relationships and sharing fish with others.

The ‘negative aspects of FADs’ categories included: no negatives; creating arguments within the family and community, less support for household activities (particularly gardening but also childcare and firewood collection), reduced attendance at church, reduced community work (including general community, church and school related activities) and other problems, such as competition with other fishers, stealing canoes, and reluctance to share resources and knowledge.

## Results

### Number of fishers and vessel type

When FADs were in the water, two main types of vessels were used for fishing: dugout canoes (paddle powered wooden dugout canoes) and fibre canoes (small fibre-glass boats often without outboard engines). Dugout canoes were by far the most important vessel type across all study sites, with 1315 hours in total recorded at FAD and 4698 hours at non-FAD fishing areas (Table 3). Only 20 hours fishing at FADs by fibre canoes were recorded versus 295 hours at non-FAD fishing areas. Nearshore FADs were almost exclusively fished by men, with only three hours of FAD fishing recorded by women, when they were part of a mixed-sex crew. The proportion of fishers that targeted nearshore FADs (as a percentage of the total unique FAD and non-FAD fishers recorded) was highest at Village D (75.5%) and Village C (54.7%). Less than 40% of fishers were recorded as fishing the FADs at Village A and Village B (Table 3). A small number of fishing events at non-FAD sites were shore-based.

**Table 3:** Total fishing hours (by vessel type), number of fishers recorded at FAD and non-FAD fishing areas and proportion of FAD fishers for each of the study villages.

		Fishing time (hours)			Number of fishers (n)	Proportion of FAD fishers (%)
		Dugout	Fibre	Shore fishing		
Village A	FAD	40.5	3.3	0	17	35.4
	non-FAD	1095	13.5	0	48	
Village B	FAD	105	5	0	23	38.3
	non-FAD	716	57.7	42.5	56	
Village C	FAD	374	0	0	47	54.7
	non-FAD	1823	2	29.0	75	
Village D	FAD	795	12	0	56	75.7
	non-FAD	1064	222	4	42	
Total	FAD	1315	20.3	0	143	64.7
	non-FAD	4698	295	75.5	221	

### Diversity of fishes caught at FADs and non-FAD sites

The diversity and number of fish caught (categorized as pelagic or benthic (reef, deep or river fishes)) at each village for FAD and non-FAD fishing sites are shown in Table 4. Diversity of fishes was calculated using the Shannon index (*H*) and species richness *(S)* standard measures [22], [23]. Unsurprisingly, pelagic fishes dominated catches from nearshore FADs in all four villages, comprising 71% of all fish caught at Village A, 55% at Village B, 78% at Village C, and 83% at Village D. The benthic fish caught at FAD sites were primarily deep-water fishes of the family Lutjanidae, such as *Etelis carbunculus*, *Etelis coruscans* and *Pristipomoides filamentosus*.

**Table 4:** Fish diversity (H), species richness (S) and total number (n) of pelagic and benthic (reef, deep-water and river) fishes caught from FAD and non-FAD fishing areas at the four study villages.

	Diversity of fishes (H)		Species richness (S)		Pelagic fish caught (n)		Benthic fish caught (n)	
	FAD	non-FAD	FAD	non-FAD	FAD	non-FAD	FAD	non-FAD
Village A	0.80	2.10	4	25	460	183	0	1449
Village B	1.27	3.21	14	89	645	531	8	3300
Village C	1.33	2.65	18	42	738	205	148	1605
Village D	1.34	1.85	17	21	3412	687	126	309

Diversity and species richness of fishes at all study villages was greater for non-FAD fishing areas compared to FAD fishing areas. The highest diversity and richness of non-FAD fishes was observed at Village B, reflecting the larger proportion of reef fish caught at non-FAD fishing areas in this village. The diversity of fishes from non-FAD fishing areas was lowest at Village D, as was the number of benthic fishes caught, reflecting the limited availability of reef fish habitat and the consequent importance of pelagic species to this village. The diversity of fishes caught at FADs was similar across three of the study villages (Village B, C and D), with a lower diversity recorded at Village A. Pelagic fish caught at the nearshore FADs belonged mostly to the families *Scombridae*, *Sphyraenidae* and *Carangidae*.

### Catch rates

**Figure 3:**
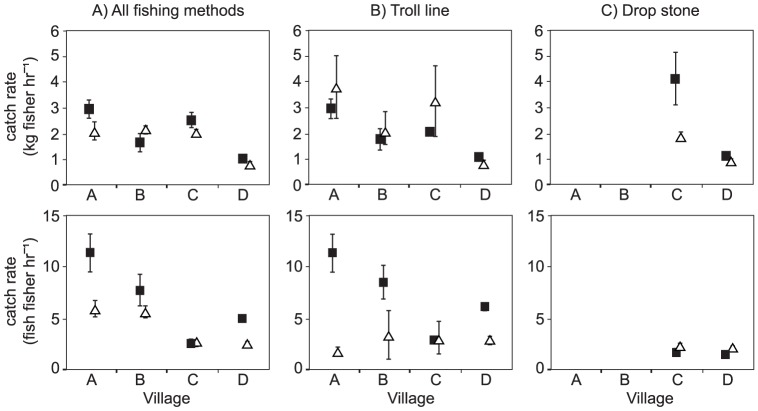
Mean FAD and non-FAD catch rates (± SE) by weight (top panel) and number of fish (bottom panel) for A) all fishing methods, B) troll line and C) drop stone fishing.

Catch rates, calculated as both weight and numbers of fish for all fishing trips recorded at FAD and non-FAD fishing areas, was compared for the four study villages (Fig. 3, Table 1). The number of fishing trips available for catch rate calculations are provided in Table 1. The mean weight-based CPUE_FAD_ ranged from 1.04 to 2.96 kg fisher^−1^ hr^−1^, and was similar to the mean CPUE_non-FAD_, which ranged from 0.87 to 2.16 kg fisher^−1^ hr^−1^. For individual villages, a significantly higher CPUE_FAD_ was evident only at Village D (Welch Two Sample t-test, t = 2.08, df = 323.7, p<0.05).

Mean catch rates based on the number of fish caught per hour ranged from 2.52 to 11.35 fish fisher^−1^ hr^−1^ at the nearshore FADs, and 0.84 to 3.79 fish fisher^−1^ hr^−1^ at non-FAD sites. CPUE_FAD_ (based on number of fish caught) was significantly higher than CPUE_non-FAD_ at two of the four villages (Village A (t = 2.72, df = 22.5, p = 0.01), and Village D (t = 6.47, df = 335.8, p<0.01)).

### Gear based catch rates

A subset of the data, containing fishing trips where only a single type of fishing gear was used, revealed more detailed information about catch rates between the FAD and non-FAD fishing at the four study villages (Fig. 3).

Sufficient records for troll line fishing were available for all four villages (Table 1). No differences in weight-based catch rates (kg fisher^−1^ hr^−1^) between FAD and non-FAD troll line fishing were observed at any of the study villages. However significantly higher catch rates based on number of fish caught per fisher hr^−1^ by troll line were observed at FADs for Village A (t = 5.11, df = 18, p<0.01) and Village D (t = 6.56, df = 196.8, p<0.01) than all other fishing areas. Overall, there was a very weak positive relationship between catch and effort for troll line fishing at the FADs (r^2^ = 0.22, p<0.05) and no significant relationship for non-FAD troll line fishing (r^2^ = −0.04, ns).

Sufficient single-method drop stone fishing trips (a mid-water fishing method targeting deeper, larger fish) were available only for Village C and D. Analysis of these data showed a significantly higher catch rate (kg fisher^−1^ hr^−1^) at Village C at FAD compared to non-FAD fishing (t = 2.1581, df = 27, p<0.05). At Village D, mean drop stone catch rates (kg fisher^−1^ hr^−1^) were slightly higher at the FAD than non-FAD but the difference was not statistically significant. For the number of fish caught by drop stone fishing, a significantly lower CPUE_FAD_ (1.49 fisher^−1^ hr^−1^) compared to CPUE_non-FAD_ (2.08 hr^−1^) was observed at Village D (t = −2.9, df = 82, p<0.01), with no significant differences at Village C.

### Annual fish catch and consumption

Estimated total annual fish catch ranged from around 4300 to 12 000 kg across study villages with an average annual catch of 7500 kg (Table 2). Nearshore FADs contributed 31 to 45% of the total annual catch, providing on average 2800 kg of fish across the four study villages. The estimated annual quantity of fish caught from FADs was substantially higher at Village C (4290±189) and Village D (3780±69) where the proportion of fishers that utilized the FADs was also higher. For three of the villages (Village A, B and C) the majority of fish caught at the nearshore FADs were kept for household consumption (75 to 85% of all fish caught). At Village D however, only 40% of fish caught at the FAD were consumed by fisher families, with the remainder mostly sold at markets (a very small proportion of fish were used for bait). Overall, these estimates suggest that FADs had the potential to contribute between 26 and 58% of the fish consumed annually across the study villages, with Village D having the greatest contribution from FADs (even with 60% of the catch sold).

### Villager perceptions of nearshore FADs

**Figure 4:**
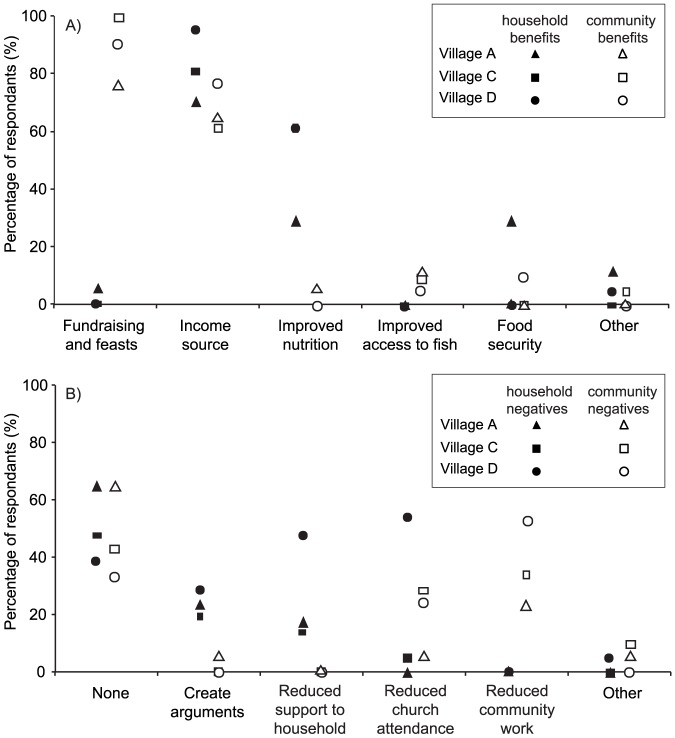
Perceived household and community benefits (graph A) and negative (graph B) aspects of the nearshore FADs mentioned by respondents during key informant interviews at three of the study villages (Villages A, C and D).

Perceived benefits of nearshore FADs were relatively uniform across the three villages (villages A, C and D) where detailed key informant interviews were undertaken (Fig. 4). Most respondents cited benefits of nearshore FADs as providing a source of family income (through the sale of fish) and improving nutrition (through an increase in fish consumption). Increased fish consumption in particular was noted at Village C and Village D (the two villages where FADs made the highest contribution to fish consumption). The main community benefits of the FADs, as perceived by the key informants, were the provision of fish for fundraising and feasts (for funerals, weddings, church and community events) and as a source of income for community related expenses (e.g. church and schools). There were other positive social dimensions mentioned including promoting sharing (e.g. fishing knowledge, new fishing methods, sharing of fish between families and households) and cementing relationships (both with other fishers and between communities through bartering and fish marketing). None of the respondents mentioned that there were no benefits from the FADs.

Some people perceived FADs as having negative impacts on families and communities, although at least 30% of respondents within each village stated there were no negative aspects of the FADs to households or the community. At the family level FADs were said to create arguments between husbands and wives (mostly attributed to husbands spending more time fishing and less time assisting with household activities). Reduced fisher support to household activities, in particular gardening, was noted at all three villages, with almost half of key informants at Village D identifying this as an issue. At the community level, the most commonly mentioned negative aspect of the FADs was a reduction in fishers' attendance at church and community activities.

## Discussion

Although Solomon Islands was among the first countries in the region to adopt offshore FADs in the industrial fishing sector [11] nearshore FADs in their modern form remain a relatively new intervention for most rural villages. The study communities had no prior experience with nearshore FADs and only fishers from Village C regularly travelled >8 km, often by paddle canoe, to fish industrial FADs located off the coast. Despite being a new concept, 35% to 75% of fishers surveyed in the four study villages fished the nearshore FADs. The apparent appeal of FADs was supported by generally positive reflections from key informants on the contribution of FADs to increasing income and consumption of fish, and as an important source of fish for community fundraising and feasts. The estimated 1300 to 4300 kg of fish that the FADs provided annually (31 to 45% of the total catch in the villages) highlights the potential role that FADs might play in food security by providing communities with access to a ‘new’ or otherwise underutilized source of fish.

Contrary to expectations, catch rates for all fishing methods were not consistently higher at the FADs compared to non-FAD fishing areas, suggesting that in general, fishing at the nearshore FADs was not necessarily more efficient than existing fishing grounds. Only at Village D were catch rates significantly higher at the nearshore FAD both in terms of weight of catch and number of fish caught. Similarly, for troll line fishing specifically (the most common gear used for fishing FADs across all sites) no significant differences were observed between weight-based catch rates at FAD and non-FAD fishing areas, although higher catch rates (in numbers of fish) were observed from FADs at Villages A and D. This result contrasts with other studies that have identified greater CPUE for troll line fishing at FADs compared to non-FAD open water fishing grounds [5], [10], [16]. Catch rates cannot be compared among these studies as Sharp [5] reported CPUE as kg vessel^−1^ hour^−1^ and Anderson and Gates [10] and Sims [16] reported CPUE as kg line^−1^ hr^−1^ and this study reports CPUE as kg fisher^−1^ hr^−1^. Given the dearth of catch rate data for nearshore FADs in the Pacific region, a regional approach to enable to comparison of data would be of value.

The lack of higher catch rates (weight based) at the nearshore FADs in this study may be explained by several factors. Firstly, schools of skipjack tuna and other small fishes tend to be found closer to FADs and near the surface, while larger fish such as yellowfin and big eye tuna tend to be found at deeper depths and further away [11], [20]. As a consequence, fishing gears such as troll lines may not the most efficient method to catch larger fish at nearshore FADs and reiterate findings by SPC that FAD catches may be limited by the availability and knowledge of fishing gears and techniques that target larger fish [19], [21].

Village-based training workshops on FAD fishing techniques were undertaken only at Village C and D and only these two villages had records of using the drop stone fishing method. At Village C, 146 non-FAD and 27 FAD fishing trips were recorded using the drop stone method and a significantly higher weight-based catch rate was identified when it was used. In the other two villages, although two community members joined a training workshop with SPC, village level training of fishers was not provided and there were no records of fishers using such mid-water fishing gears. Village-based training of fishers (using methods suitable for boats and gears available to rural fishers), sharing knowledge between villages and drawing on lessons learned by fishers should to be taken into consideration within a Solomon Islands national FAD program.

A second contributing cause to the lack of higher troll line catch rates around FADs is that catching fish in the open ocean, generally by targeting schools of pelagic fish when they are visible on the surface [24], requires great skill and deep understanding of the currents and boundaries between different bodies of water. Catches from such talented fishers are not necessarily correlated with the length of time fished. Less skill is required for troll line fishing close to a FAD. This is exemplified by the higher variation in CPUE_non-FAD_ for troll line fishing and the lack of any relationship between catch and effort for troll line fishing, particularly for the non-FAD fishing areas (FAD r^2^ = 0.22, non-FAD r^2^ = −0.04). Tuna caught from free swimming tuna schools are also generally larger than those caught at FADs [20].

Thirdly, the productivity and diversity of existing fisheries at some sites may be greater than the productivity of pelagic fishes. For example, Village D had the greatest proportion of FAD fishers and the highest annual proportion of fish derived from FADs, yet this site had the lowest catch rates for both FAD and non-FAD fishing. The diversity of non-FAD fishes was lowest at this site, reflecting the limited reef availability and importance of pelagic fishes to this community. Conversely, the villages with the least annual contribution of fish derived from the FADs (villages A and B), and least proportion of fishers targeting FADs, had relatively higher catch rates for both FAD and non-FAD fishing and a greater diversity of non-FAD fishes. These results have important implications for nearshore FAD site selection in Solomon Islands, indicating that villages that experience low catch rates, have limited diversity of fishes or have degraded reef fisheries have a greater likelihood of using FADs to better effect.

This study is the first of its kind for Solomon Islands, a nation for which it has been projected that coastal fisheries will not be able to supply the fish needed to meet increasing demand without improved coastal fisheries management and alternative sources of fish [4]. With a focus on food security benefits and social dimensions of nearshore FADs for subsistence based rural coastal communities, this study provides evidence that nearshore FADs can contribute to increasing the supply of fish to coastal communities in Solomon Islands.

Along with their role in securing an adequate supply of fish in the region, nearshore FADs have also been widely promoted as having a role as a fisheries management tool (through the transfer of fishing effort from the reef to pelagic and oceanic resources) and as a climate change adaptation measure [6], [8], [9]. While it is clear that FADs increase the supply of fish, it is not possible from this analysis to determine whether their presence reduced pressure on existing reef fisheries as postulated by others [1], [10]. Similarly, data limitations restricted the analysis of nearshore FADs as a fisheries management tool in a three-year study in the Cook Islands [13], [25], [26]. Further analysis is needed if FADs are to evolve beyond their current potential; if not FADs will join other ‘livelihood diversification’ options as much-touted but largely untested contributors to improved coastal fisheries.

This study has highlighted that nearshore FADs can have negative impacts on village life. A reduction in the time that male fishers spend on other household (in particular gardening) and community activities may have long-term impacts for households and communities if not acknowledged and addressed. Several fishers from Village D, for example, fished the nearshore FAD every day; and stated they were “*extremely happy with the catch*” (*pers comm.* Village D fisher). The fishers started selling and trading fish with inland communities and as they were spending more time fishing, they were spending less time in their gardens. After nearly six months their nearshore FAD was lost; and the fishers lost access to a fishing area that they had come to rely upon for their livelihoods and their neglected gardens were in disarray. This was a harsh lesson for the families who needed to rebuild their gardens and, in the meantime, rely on a limited supply of garden produce and fish from less productive fishing grounds. Similar observations have been made around the béche-de-mer fishery in Solomon Islands where a concentration of effort on béche-de-mer fishing at the expense of other livelihood activities [27] resulted in a period of hardship when a national ban was imposed on the fishery, while gardens and other livelihoods were being rebuilt [28].

While not strictly using a livelihoods approach [29]–[32] we have taken a livelihoods perspective in assessing the contribution FADs could make to improved food security. Clearly, FADs easily fit into the dynamic nature of daily village life, introducing a new livelihood option for rural households already adept at fishing. The ease with which people move between garden-based livelihoods and fishing is typical of rural Solomon Island life, and FADs may be seen as just another adaptation in the portfolio of options. Although we conclude FADs can increase the supply of fish, the transient nature of FADs brings risks from reliance on them, reduced time spent on other more secure but less profitable livelihood options such as gardening, and the potential for intra-household conflict.

Drawing on the findings of this study, and building on insights from earlier papers, a range of evidence-based conclusions begin to emerge about the implementation of nearshore FADs. As Solomon Islands pursues development of a national FAD program, the technical aspects of deployment to maximize FAD longevity, such as site selection and the design of the FAD, will be critical. The experience of villagers will continue to augment expertise from SPC and bring new innovations in design, maintenance and redeployment. More difficult, but equally important to realizing the potential for nearshore FADs, will be the institutional aspects of their use. Communities need the information and space to assess the likely benefits and trade-offs needed to manage the introduction of such a livelihood opportunity.

In this respect, nearshore FADs need to be embedded in the wider development planning of communities and national agencies in order to recognize benefits and tradeoffs, including those which disproportionately affect some members of society, such as women gardeners, and to be able to plan for these accordingly. At national scales, effort should focus on more food ‘insecure’ communities that have a high reliance on fish and limited access to diverse or productive fishing areas.

At the scale of communities, the interplay between use rights and the many dimensions of customary tenure and ownership [33]–[35] will be a key determinant of success. The vandalism of FADs that is common in Solomon Islands and in the Pacific region more generally [12], [14], [17] reveals not only issues around the distribution of direct benefits from FADs, but also highlights the utility of FADs as assets and levers in broader tenure disputes.

Finally, FADs are widely promoted as tools in biodiversity conservation and fisheries management. Their promise lies in augmenting livelihoods and the supply of fish while management measures reliant on not catching fish or catching fewer or larger fish are implemented. Typically there is a lag between initiating such interventions and the flow of benefits from them. Empirical tests of the efficacy of FADs in doing more than just increasing the supply of fish will require their integration into national, provincial and community development plans (see also [8]). In short, and unsurprisingly, although FADs can supply more fish, they are not a technical panacea that will ensure more resilient livelihoods for rural Solomon Islanders. Rather, FADs remain potential contributors to broad governance and management pathways to rural development, but this proposition remains largely untested.

## Supporting Information

S1 Table **Nearshore FAD design, deployment depth, FAD longevity, distance from village to the FADs and distance from village to other fishing areas (median) for each of the FADs deployed at the four study sites.**
(DOCX)

S2 Table **Population size, number of households and Information on the key informants interviewed at the four study villages.**
(DOCX)

S1 File **Nearshore FAD design and location.**
(DOCX)
