# Supplementary material for: The Contribution of Nearshore Fish Aggregating Devices (FADs) to Food Security and Livelihoods in Solomon Islands
Source: PLoS One. 2014 Dec 16;9(12):e115386. doi: 10.1371/journal.pone.0115386 (PMC4267842; doi:10.1371/journal.pone.0115386)
Supplement: S1 Table — Nearshore FAD design, deployment depth, FAD longevity, distance from village to the FADs and distance from village to other fishing areas (median) for each of the FADs deployed at the four study sites. (DOCX) [file pone.0115386.s001.docx]

**Table S1. Nearshore FAD design, deployment depth, FAD longevity, distance from village to the FADs and distance from village to other fishing areas (median) for each of the FADs deployed at the four study sites.**

|  | **FAD deployment^a^** | | **FAD design** | **FAD depth (m)** | **FAD longevity (days)** | **Distance to FAD (km)** | **Distance to other fishing areas (km)** |
| --- | --- | --- | --- | --- | --- | --- | --- |
| Village A | 1 |  | C | 450 | 15 | 4.5 | 2.7 |
|  | 2 |  | D | 450 | 110 | 2.7 |  |
| Village B | 1 | A | B | 410 | 279 | 1.0 | 2.8 |
|  |  | B | B | 430 | 293 | 3.9 |  |
| Village C | 1 | A | C | 340 | 272 | 2.0 | 2.2 |
|  |  | B | C | 330 | 272 | 7.3 |  |
| Village D | 1 |  | A | 265 | 39 | 7.3 | 5.1 |
|  | 2 |  | A | 260 | 129 | 7.3 |  |
|  | 3 |  | D | 270 | 158 | 7.3 |  |

^a^ FADs deployed on separate occasions are denoted numerically, A and B ref to two FADs deployed off the one village.
