# Supplementary material for: The Contribution of Nearshore Fish Aggregating Devices (FADs) to Food Security and Livelihoods in Solomon Islands
Source: PLoS One. 2014 Dec 16;9(12):e115386. doi: 10.1371/journal.pone.0115386 (PMC4267842; doi:10.1371/journal.pone.0115386)
Supplement: S2 Table — Population size, number of households and Information on the key informants interviewed at the four study villages. (DOCX) [file pone.0115386.s002.docx]

**Table S2 Population size, number of households and primary livelihood activities of key informants interviewed at the four study villages.**

|  | Village A | Village B | Village C | Village D |
| --- | --- | --- | --- | --- |
| **Village Information** | | | | |
| Population | 416 | 122 | 341 | 284 |
| Number of households | 58 | 17 | 45 | 42 |
| **Respondents interviewed** | | | | |
| Total | 17 | n/a | 21 | 21 |
| Female | 2 | n/a | 3 | 5 |
| Male | 15 | n/a | 16 | 16 |
| **Primary livelihood activity (% of respondents)** | | | | |
| Garden | 18 |  | 42 | 24 |
| Fishing | 70 |  | 57 | 67 |
| Other^a^ | 12 |  | 1 | 9 |

^a^ Other livelihood activities included labour, teaching and carpentary
