# Supplementary material for: The Contribution of Nearshore Fish Aggregating Devices (FADs) to Food Security and Livelihoods in Solomon Islands
Source: PLoS One. 2014 Dec 16;9(12):e115386. doi: 10.1371/journal.pone.0115386 (PMC4267842; doi:10.1371/journal.pone.0115386)
Supplement: S1 File — Nearshore FAD design and location. (DOCX) [file pone.0115386.s003.docx]

**File S1 Nearshore FAD design and location**

The broader objectives of this study were to assess the longevity of different anchored FAD designs. In total 22 FADs were deployed at 13 locations in nearshore waters of Solomon Islands. FAD design was based on the SPC Indian Ocean Design [[1](#_ENREF_1)], with several variations.

Design A: Flotation device comprising of a series of pressure and purse seine floats with a combination of stainless steel cable (to reduce line damage at the surface), polypropylene rope (12 mm) and nylon rope (20 mm) to create a caternary curve in the rope, anchored with cement drums or heavy machinery and grapnel anchor.

Design B: Flotation device comprising of a series of pressure and purse seine floats with a combination of polypropylene rope (20 mm) and nylon rope (20 mm) to create a caternary curve in the rope, anchored with cement drums or heavy machinery.

Design C: Flotation device comprising of a series of pressure and purse seine floats, using polypropylene rope (20 mm) anchored with cement drums or heavy machinery and grapnel anchor.

Design D: Flotation devise comprising of locally sourced materials (eg bamboo) anchored with cement drums or heavy machinery and grapnel anchor.

FAD designs utilized at the study villages are provided in Table S1.

**Reference**

1. Chapman L, Pasisi B, Bertram I, Beverly S, Sokimi W (2005) Manual on fish aggregating devices (FADs): Lower-cost moorings and programme management. Noumea: Secretariat of the Pacific Community.
